# Case Report: A rare case of spontaneous lesser omental sac hernia with pneumomediastinum

**DOI:** 10.3389/fsurg.2026.1900310

**Published:** 2026-10-13

**Authors:** Jianlei Yang, Zili Zhang

**Affiliations:** 1 Department of Surgery, The Third Central Hospital of Tianjin, Tianjin, China; 2 Tianjin Institute of Hepatobiliary Disease, Tianjin, China

**Keywords:** gastric outlet obstruction, lesser omental sac herniation, Macklin effect, case report, pneumomediastinum

## Abstract

Pneumomediastinum typically results from trauma, iatrogenic injury, or an acute rise in intrathoracic pressure, as occurs with forceful coughing or vomiting. Here, we report an adolescent male patient with a congenital lesser omental defect, which led to small bowel herniation into the lesser sac. The incarcerated intestinal loop compressed the pylorus, resulting in gastric outlet obstruction, intractable vomiting, and subsequent pneumomediastinum. This case illustrates lesser sac herniation leading to vomiting-induced pneumomediastinum via the “Macklin effect”.

## 1 Introduction

The lesser sac (synonymous with omental bursa) is the peritoneal space posterior to the stomach and lesser omentum (1). Lesser sac hernias, a rare form of intra-abdominal hernia, occur when abdominal contents protrude through a defect or weakness in the lesser sac wall (Figure 1) (2). Its clinical features are nonspecific, mainly including abdominal pain, distension, nausea, vomiting. Pneumomediastinum is defined as free air or gas located within the mediastinum (3). Its common etiologies encompass trauma and iatrogenic injury or precipitating factors, such as coughing, vomiting, sneezing, strenuous exercise, seizures, diving, vaping, and toxic gas inhalation (4). Vomiting has been documented as the triggering factor in approximately 8% of cases (5). Herein, we report a rare case of spontaneous lesser sac hernia complicated by secondary pneumomediastinum following severe vomiting.

**Figure 1:**
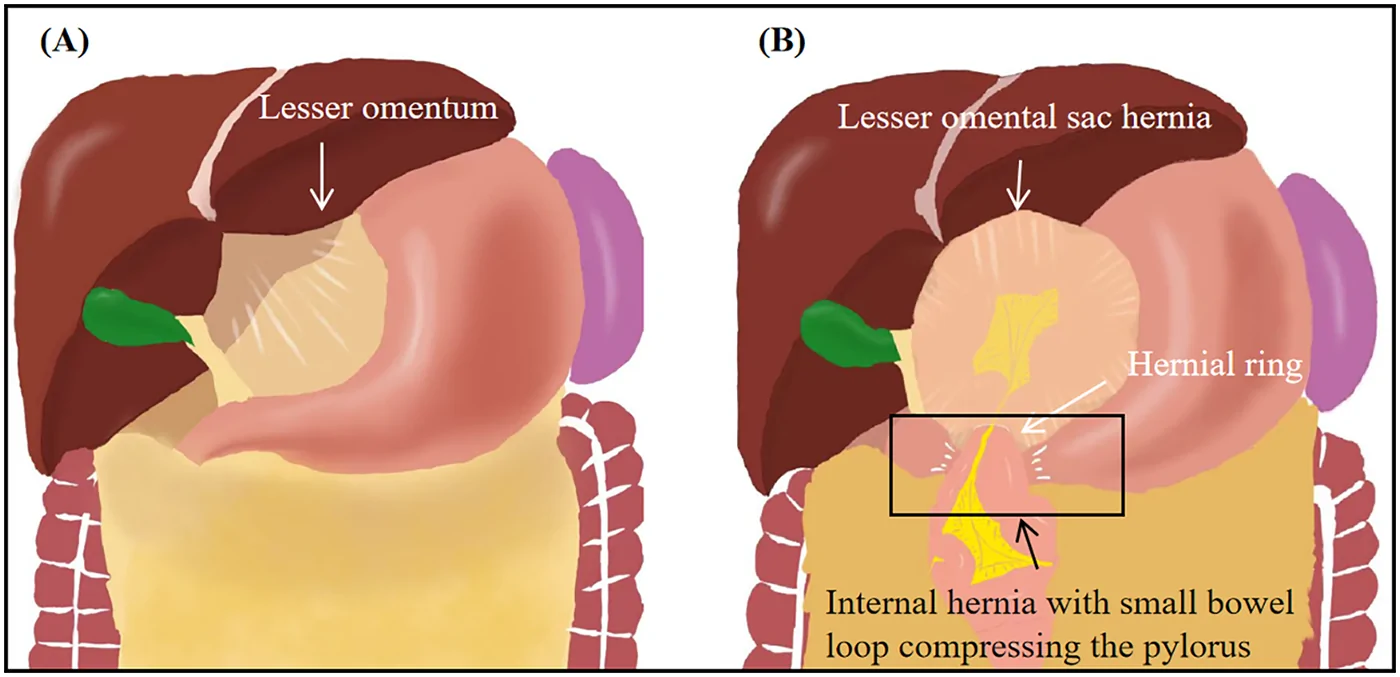
Schematic diagram nof pyloric obstruction induced by congenital lesser sac hernia. **(A)**: Schematic diagram of the lesser omentum. **(B)**: Schematic diagram of pyloric obstruction secondary to pyloric compression by incarcerated small intestinal loops from an internal hernia.

The case report follows the latest practice of surgical case reporting (6).

## 2 Case description

An 18-year-old male high school student, Body mass index (BMI): 20.9 kg/m², presented with acute epigastric pain, nausea, and vomiting 2 h postprandium. He reported no recent abdominal trauma, strenuous exertion, smoking, or vaping, and had no significant prior medical history. Vital signs were stable on admission. Table 1 summarizes the patient’s clinical timeline in detail. Physical examination showed epigastric fullness with mild tenderness and normoactive bowel sounds. Laboratory tests revealed leukocytosis (11.32 × 10⁹/L). Upper abdominal Computed Tomography (CT) (Figure 2) demonstrated: 1. Massive gastric distension; 2. Dilated small bowel loops between the liver and stomach; 3. Peri-esophageal gas collection in the inferior mediastinum. In the emergency room, the patient suffered from worsening nausea and vomiting again. Urgent chest CT and abdominal contrast-enhanced CT were performed. Chest CT (Figure 3) showed gas collection between the esophagus and pericardium, with cephalad extension surrounding the left common carotid artery. Abdominal contrast-enhanced CT (Coronal Multiplanar Reformation) (Figure 4): Small bowel loops traverse anterior to the transverse colon and pylorus, herniating into the lesser sac. The herniated bowel displaces and compresses the collapsed gastric cavity. No small bowel is visualized in the lower abdomen or pelvis. These findings are diagnostic of a lesser sac hernia. No free intraperitoneal gas is present.

**Table 1:** Timeline of the episode of care.

Time point	Event and key findings
2 h after a meal	Acute epigastric pain, nausea and vomiting; no trauma, strenuous exertion, smoking or vaping.
On admission	Stable vital signs; epigastric fullness, mild tenderness; WBC 11.32 × 10⁹/L. Upper abdominal CT: marked gastric distension, dilated small bowel between liver and stomach, peri-esophageal gas in the lower mediastinum.
Emergency Room observation	Vomiting worsened. Chest CT: mediastinal gas between esophagus and pericardium extending to the left common carotid artery. Contrast CT: small bowel herniating into the lesser sac and compressing the pylorus; no free intraperitoneal air.
Before surgery	Upper Gastrointestinal contrast study: no extravasation; complete antral obstruction from external compression.
Emergency laparoscopy (same day)	3-cm congenital lesser-omental defect; ∼140 cm of ileum herniated (incarcerated segment ∼130 cm proximal to the terminal ileum); band-like inflammatory changes ∼75 cm beyond the hernia ring; bowel viable; negative air-insufflation test; thin, translucent lesser omentum.
Postoperative day 6	Normal diet resumed; discharged without complication.
1–24 months	Symptom-based outpatient follow-up; no recurrence of hernia or pneumomediastinum over 2 years.

**Figure 2:**
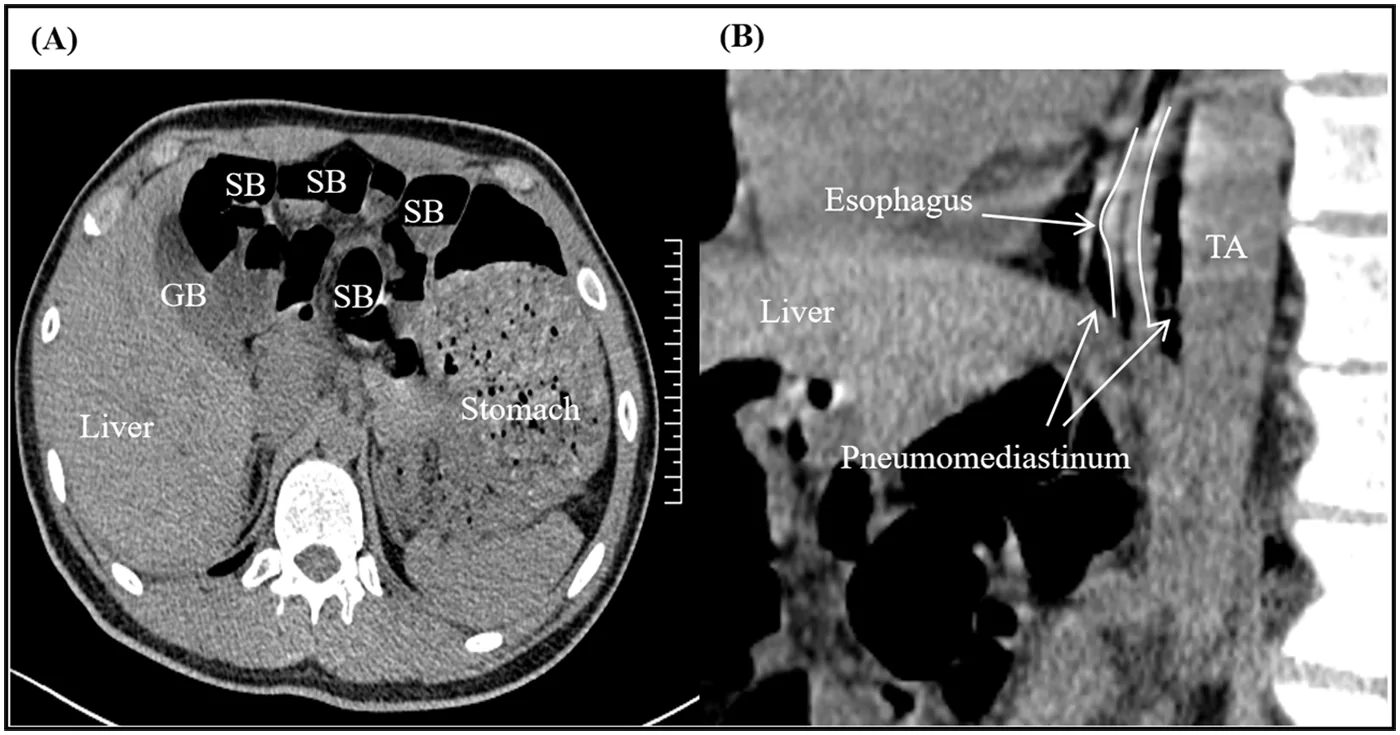
Upper abdomen computed tomography, axial view **(A)**, sagittal view**(B)**. **(A)**: Marked gastric distention with retained contents; abnormal accumulation of SB (Small Bowel) between the liver and stomach with dilatation; GB: Gallbladder. **(B)**: Peri-esophageal gas collection in the inferior mediastinum; TA: Thoracic Aorta.

**Figure 3:**
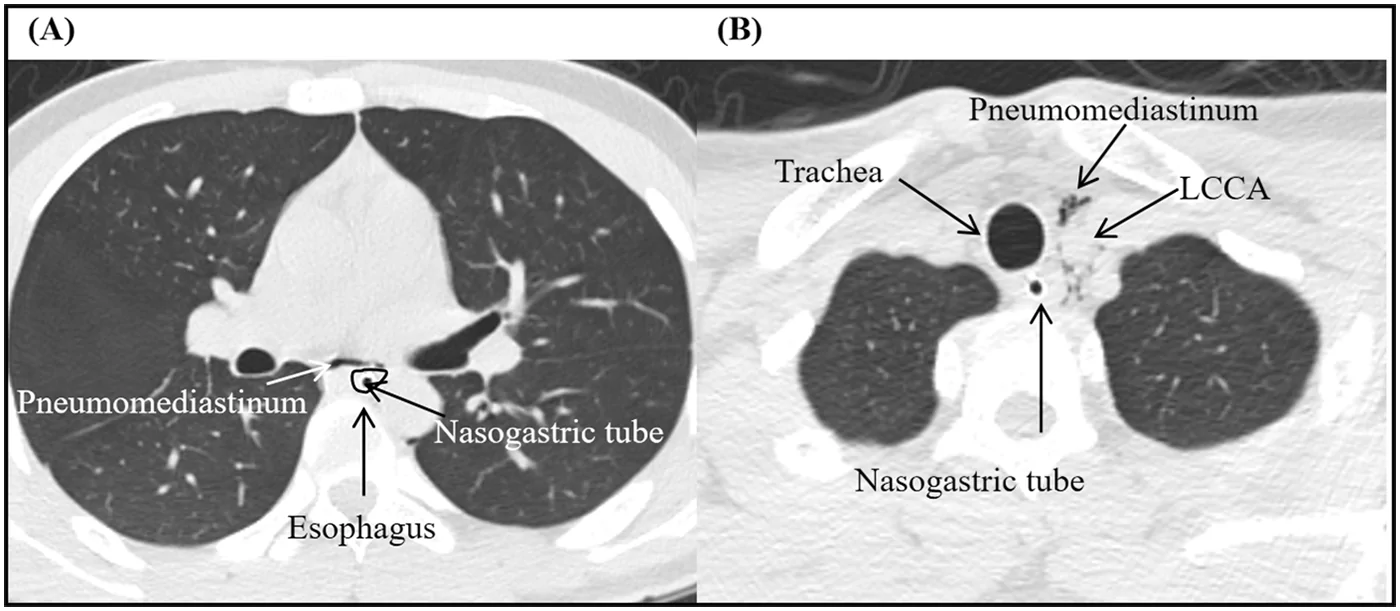
Chest computed tomography, axial view. **(A)**: Air accumulation between esophagus and pericardium. **(B)**: Gas spreading up to the LCCA (Left Common Carotid Artery).

**Figure 4:**
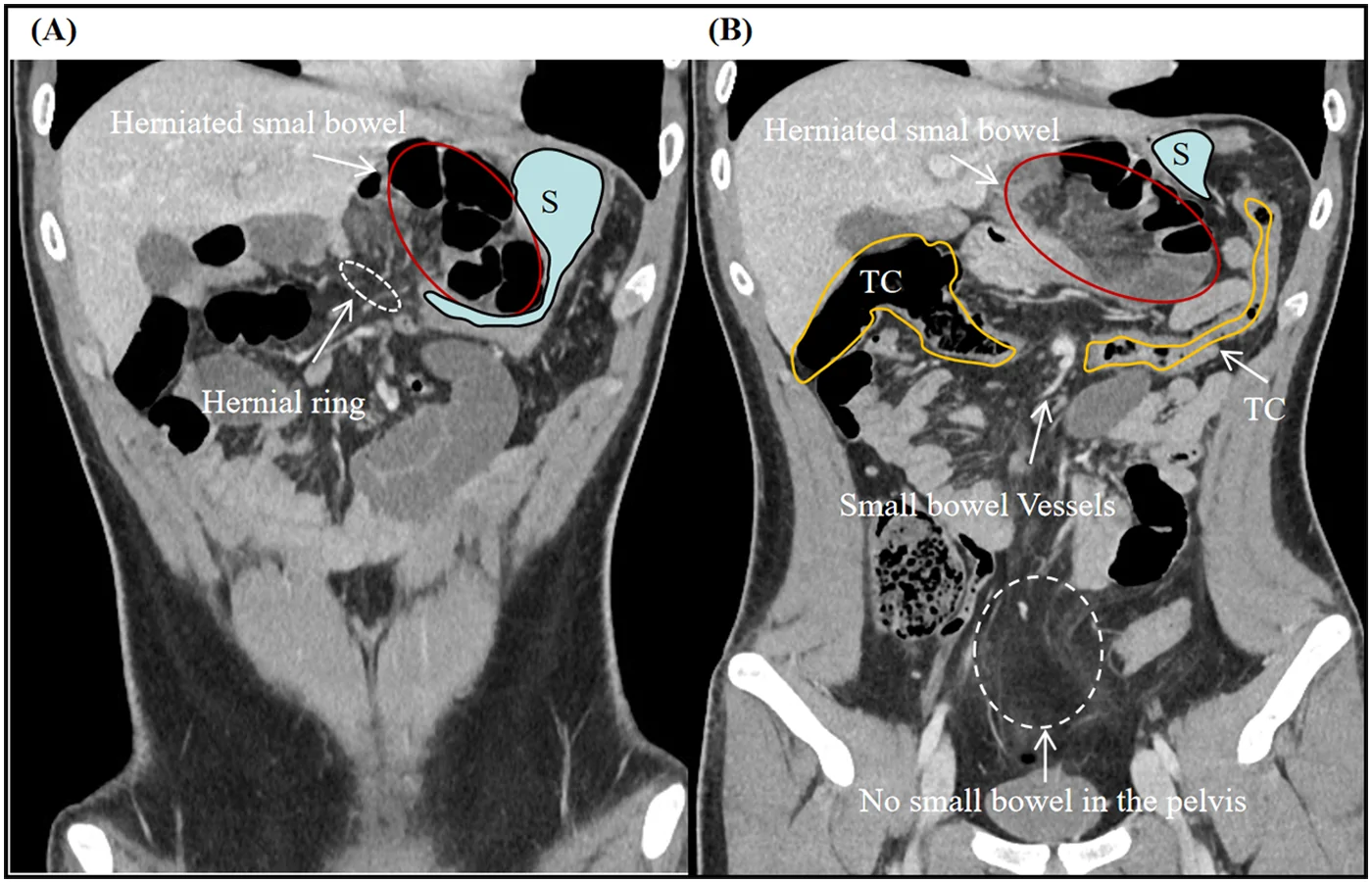
Abdomen contrast-enhanced computed tomography, coronal view. **(A)**: Small bowel mesentery enters into the lower omental sac (Red-Solid-Line Marked Area) through the hernia ring (Dashed-line area) to form a small omental sac hernia; and compresses the S (stomach). **(B)**: Ascending small bowel mesenteric vessels (arrow) can be seen in front of the TC (Transverse Colon), and the lower abdomen and pelvic cavity (Dashed-line area) are devoid of small bowel loops.

Upper gastrointestinal contrast study (Supplementary Figure S1) was performed: no contrast extravasation but revealed complete antral obstruction due to extrinsic compression. Thoracic surgery consultation postulated that the pneumomediastinum was likely associated with severe retching and vomiting, and esophageal perforation could not be definitively excluded. Preoperative diagnosis: 1. Lesser sac hernia with gastric outlet obstruction; 2. Mediastinal free air of undetermined cause, esophageal perforation pending exclusion.

Due to escalating pain, emergency laparoscopy was performed. Approximately 140 cm of the ileum had herniated through the 3-cm congenital defect in the lesser omentum into the lesser sac, with the incarcerated segment lying approximately 130 cm proximal to the terminal ileum (Supplementary Figure S2C). The herniated loop compressed the pyloric, causing gastric outlet obstruction (Supplementary Figure S2A). The shape and color of the small bowel at the hernia ring were normal; approximately 75 cm distal to the hernia ring, the herniated small bowel and mesentery exhibited band-like inflammatory changes as a result of incarceration (Supplementary Figure S2B), though viability was preserved. To determine the cause, the lesser omentum was fully opened, and it was confirmed that there were no signs of trauma or inflammatory adhesions within the omental sac.

Continued exploration of the hiatus, lower esophagus, cardia with air insufflation test via nasogastric tube (Supplementary Figures S2D, E), duodenum, and remaining viscera showed no perforation or pathology. Focal areas of the lesser omentum appeared thin and translucent (“cicada-wing” appearance, Supplementary Figure S2F). The operation was ended after indwelling drainage at the lesser omental sac. Post-operative diagnosis: lesser sac hernia, secondary pneumomediastinum, pyloric obstruction. The patient recovered uneventfully, resumed a normal diet by postoperative day 6, and was discharged. Follow-up was symptom-based through outpatient visits at 1, 3, 6, 12, and 24 months without routine surveillance CT, and imaging would be repeated only if obstructive symptoms recurred. No recurrence of internal hernia or pneumomediastinum was documented during the 2-year follow-up period.

## 3 Discussion

Transomental hernias are exceedingly rare, constituting 1% to 4% of all intra-abdominal hernias (7), with most reported cases occurring in patients over 50 years of age (8). Lesser sac defects may result from congenital developmental anomalies associated with an elongated small bowel mesentery, intestinal malrotation, and anomalous peritoneal attachments, as well as acquired factors including abdominal trauma, chronic inflammation, and low BMI (8, 9). Of note, the incidence of postoperative lesser sac hernias has risen gradually in recent years, attributable to the increasing popularity of abdominal surgeries, especially bariatric procedures such as Roux-en-Y gastric bypass (10). Additionally, elevated intra-abdominal pressure and accelerated intestinal peristalsis serve as major precipitating factors for this condition. The patient’s internal hernia was classified as spontaneous based on these key findings: 1. No surgical history or abdominal trauma. 2. Low body mass index (BMI 20.9 kg/m²) with reduced visceral fat and attenuation of the lesser omentum to a translucent (“cicada-wing” appearance, Supplementary Figure S2F), increasing vulnerability to injury (11).

The mediastinal free air required differentiation between an occult gastrointestinal perforation and pneumomediastinum induced by vomiting. A perforated, strangulated internal hernia with air ascending from the peritoneal cavity was considered first, but was not supported: abdominal CT showed no free intraperitoneal air; the intact esophageal hiatus keeps the mediastinum separate from the peritoneal cavity; and no retroperitoneal perforation or free air was seen on imaging or at operation, although retroperitoneal emphysema can reach the mediastinum through the aortic hiatus. Forceful vomiting can itself rupture the distal esophagus (Boerhaave syndrome) (12), this was likewise excluded here, as the upper gastrointestinal contrast study showed no extravasation (Supplementary Figure S1A), direct inspection and the air-insufflation test were negative (Supplementary Figures S2D, E), and neither mediastinal effusion nor inflammatory exudate—which would be expected after repeated vomiting against a perforation—was present. Conversely, the findings are more consistent with vomiting-induced pneumomediastinum via the “Macklin effect”.Vomiting-induced pneumomediastinum typically arises via two mechanisms (3): First, the “Macklin effect”: vomiting causes diaphragmatic elevation, increased pulmonary pressure leading to alveolar rupture, and subsequent gas migration to the mediastinum through peribronchial and perivascular sheaths. Second, esophageal rupture may occur due to vomiting, which was excluded.

In this case, pneumomediastinum secondary to lesser omental sac herniation may developed through the following mechanism (Supplementary Figure S3): Internal herniation of small bowel loops compressed the pyloric region, causing gastric outlet obstruction. Progressive gastric distension compressed the lesser sac, worsening bowel loop incarceration and intensifying abdominal pain—a potent vomiting stimulus. Simultaneously, pyloric obstruction retained gastric contents, generating supraphysiologic pressure in the proximal stomach. Forceful retrograde peristaltic contractions amplified emetic forces, culminating in pneumomediastinum via the “Macklin effect”.

According to the latest literature review (11), 31 cases of lesser sac hernia have been documented in English-language publications from 1946 to 2023. Vomiting occurred in 61.3% (19/31) of the patients (11). Notably, none of the 31 cases developed pneumomediastinum (2, 11, 13–41). Excluding cases with upper abdominal surgery or trauma history, 25 spontaneous lesser sac hernias were identified (Table 2), with a mean age of 44 years. The most common anatomical defect was lesser omental deficiency (96%, 24/25), followed by gastrocolic ligament defects (40%, 10/25), transverse mesocolon defects (16%, 4/25), and foramen of Winslow (12%, 3/25). CT clearly demonstrates abnormal bowel distribution and provides key evidence to confirm lesser sac hernia. Of the 25 cases, seven received a definitive preoperative diagnosis on CT scans. The rate of small bowel resection was 33% (5/15) in patients who underwent preoperative CT, which was markedly lower than the 50%(5/10) resection rate observed in patients without preoperative CT. Despite this benefit in reducing bowel resection risk, preoperative CT imaging should not lead to delayed surgical intervention.

**Table 2:** Selected articles reporting spontaneous lesser sac hernia.

Type of anatomical variation	Studies	Age	gender	CT	Preoperative diagnosis of lesser sac hernia	Bowel resection
Lesser omental defect	Baek, 1994 (18)	88	F	Yes	No	Yes
	Coulier, 2007 (19)	79	F	Yes	Yes	Na
	Duarte, 2002 (20)	36	M	NO	No	Yes
	Joao, 2022 (21)	44	M	Yes	No	Yes
	Aylett, 1946 (22)	34	M	No	No	No
	Rathnakar, 2016 (23)	54	M	No	NO	No
	Tran, 1991 (24)	24	F	Yes	Yes	Yes
	Alves AS, 2023 (11)	65	F	Yes	Yes	No
Transverse mesocolic defect and lesser omental defect	Bahadori, 2003 (25)	14	F	No	No	Yes
	Chou, 2005 (26)	17	F	Yes	Yes	No
	Kulkarni, 2020 (27)	54	F	Yes	NO	NO
	Kundaragi, 2014 (28)	55	M	Yes	YES	No
Lesser omental defect and Winslow	Rich, 2002 (29)	67	F	No	NO	YES
	Saida, 2000 (30)	33	M	Yes	No	No
	Tjandra, 1991 (31)	43	F	yes	No	No
Gastrocolic ligament defect and lesser omental defect	Chattapadhyay, 1982 (32)	28	F	No	No	No
	Chen, 2012 (33)	56	F	Yes	No	No
	Hanatate, 1997 (34)	45	M	No	No	Yes
	Inoue, 1996 (35)	49	M	Yes	Yes	Yes
	Li, 2017 (36)	38	F	No	No	Yes
	Min, 2010 (37)	47	F	Yes	Yes	Yes
	See, 2002 (38)	41	M	No	No	No
	Talebpour, 2005 (39)	29	M	Yes	No	No
	Yasuda, 1989 (40)	20	M	No	NO	No
Gastrocolic ligament defect	Lee, 2016 (41)	57	M	Yes	NO	NO

F, female; M, male.

Although vomiting is frequently reported (61.3%, 19/31) (11), pneumomediastinum is a rare event. To identify why it occurred in our patient, we re-examined all 31 reports (2, 11, 13–41) along two axes: the degree of internal herniation and the degree of gastric-outlet obstruction (Table 3). The extent of internal herniation was assessed based on the length of the herniated bowel, the presence of strangulation, and the size of the hernia orifice. The severity of gastric-outlet obstruction in each case was graded based on objective imaging, contrast, or operative findings as G0 (no gastric involvement), G1 (compression with a patent outlet), G2 (incomplete obstruction), or G3 (complete obstruction).

**Table 3:** Summary of internal-hernia characteristics and gastric-outlet obstruction grades.

Case	Age/Sex	Herniated viscus	Length	Defect	Grade	Pneumomedia-stinum	Bowel necrosis
Aylett 1946 (22)	34 M	Small bowel (single loop)	NR	NR	G0	No	No
Baek 1994 (18)	88 F	Ileum	∼20 cm	2 cm	G1	No	Yes
Bahadori 2003 (25)	14 F	Small bowel (two strangulated loops)	NR	NR	G1	No	Yes
Chattopadhyay 1982 (32)	28 F	Small bowel (with diverticulum)	NR	NR	G0	No	No
Chen 2012 (33)	56 F	Jejunum	∼40 cm	∼1.5 cm	G0	No	No
Chou 2005 (26)	17 F	Jejunum (80 cm distal to Treitz)	∼150 cm	NR	G0	No	No
Coulier 2007 (19)	79 F	Gastric volvulus/incarceration	-	NR	G3	No	-
Duarte 2002 (20)	36 M	Ileum	90 cm	NR	G0	No	Yes
Gants 1953 (13)	30 M	Stomach + transverse colon + omentum	-	5 cm	G2	No	No
Hanatate 1997 (34)	45 M	Ileum	140 cm	5.5 × 3/2 × 3 cm	G1	No	Yes
Inoue 1996 (35)	49 M	Ileum	50 cm	Double defect	G1	No	Yes
Joao 2022 (21)	42 M	Jejunum	∼20 cm	∼2 cm	G0	No	Yes
Jolliffe 2021 (14)	77 M	Jejunum	60 cm (ischemic)	NR	G0	No	Yes
Konishi 2014 (2)	42 M	Jejunum (10 cm distal to Treitz)	150 cm	5 cm	G0	No	No
Kulkarni 2020 (27)	54 F	Proximal small bowel	∼20 cm	NR	G2	No	No
Kundaragi 2014 (28)	55 M	Proximal jejunum	NR	5 cm/∼4 cm	G1	No	No
Lee 2016 (41)	57 M	Jejunum + proximal ileum	∼50 cm	2 cm	G0	No	No
Li 2017 (36)	38 F	Ileum (with Meckel)	∼100 cm	NR	G0	No	Yes
Liu 2020 (15)	73 F	Jejunum	∼200 cm	∼3 × 4 cm	G1	No	Yes
Masubuchi 2012 (16)	57 F	Ileum	NR	NR	G0	No	No
Min 2010 (37)	47 F	Jejunum (40 cm distal to Treitz)	∼200 cm	Double defect	G2	No	Yes
Rathnakar 2016 (23)	54 M	Jejunum	NR	3 × 2 cm	G0	No	No
Rich 2002 (29)	67 F	Cecum + right colon	-	NR	G0	No	No
Saida 2000 (30)	33 M	Ileum	∼100 cm	NR	G0	No	No
See 2002 (38)	41 M	Terminal ileum	20 cm	Two 2 × 2 cm	G0	No	No
Talebpour 2005 (39)	29 M	Jejunum	20 cm	NR	G1	No	No
Tjandra 1991 (31)	43 F	Cecum + ascending colon	-	Foramen ovale admits 3 fingers	G0	No	No
Tran 1991 (24)	24 F	Jejunum (from 30 cm distal to Treitz)	125 cm	4 cm diameter	G3	No	Yes
Ugianskis 2022 (17)	35 M	Entire small bowel + duodenum + pancreatic head	NR	Large orifice not measured	G1	No	No
Yasuda 1989 (40)	20 M	Distal half of small bowel	NR	NR	G0	No	Yes
Alves 2023 (11)	65 F	Ileum (closed loop)	NR	NR	G0	No	No
Present case	18 M	Ileum	140 cm	3 cm	G3	Yes	No

F, female; M, male; NR, not reported.

The severity of internal herniation showed no relationship with pneumomediastinum: herniated small-bowel length ranged from 20 to 200 cm (*n* = 18) and gangrenous or infarcted bowel was described in 12, including 125 cm of gangrenous jejunum (24) and approximately 200 cm of infarcted jejunum (15, 37), yet no pneumomediastinum occurred. Our patient’s 140 cm through a 3-cm congenital defect, with viable inflamed bowel and no resection, lies within this range; notably, the only other 140-cm herniation, with gangrenous ileum (34), likewise had no pneumomediastinum.

The grade-specific counts were 18 at G0, 8 at G1, 3 at G2, and 2 at G3 in the literature. By contrast, gastric-outlet obstruction behaved as a necessary threshold: no case below G3 developed pneumomediastinum (G0 *n* = 18, G1 *n* = 8, G2 *n* = 3), whereas the only event occurred in our own G3 patient; heavier obstruction impairs gastric emptying further, provoking more forceful vomiting and raising the intrathoracic pressure sharply. Necessity is also supported from outside the hernia literature, where two patients with superior mesenteric artery syndrome, a mechanical high-grade obstruction of the third part of the duodenum caused by extrinsic vascular compression, developed pneumomediastinum through intractable vomiting (42, 43). Yet complete obstruction was not sufficient on its own: the only prior small-bowel G3 (24) had a moderately distended stomach, nasogastric suction from admission and no documented retching, and the other G3 case, a gastric volvulus without herniated bowel (19), had an infarcted wall and was decompressed endoscopically and intraoperatively. Collectively, pneumomediastinum thus required high-grade gastric-outlet obstruction together with repeated forceful retching acting on a markedly distended viable stomach; the lean, young patients described with superior mesenteric artery syndrome further suggest host susceptibility as an additional contributing factor (42, 43).

Uncomplicated spontaneous pneumomediastinum usually resolves spontaneously, and routine surveillance imaging is not a fixed requirement; follow-up can be symptom-based and decided case by case (3). Our patient was followed clinically, and his guardians declined further thoracic imaging because of concern about cumulative radiation from repeated CT scans. In conclusion, lesser sac herniation may induce severe vomiting and secondary pneumomediastinum. CT serves as the preferred imaging modality for the definitive diagnosis of this rare hernia, but CT evaluation should not be regarded as a reason to delay surgical exploration.

## Data Availability

The original contributions presented in the study are included in the article/Supplementary Material, further inquiries can be directed to the corresponding author.

## Supplementary material

SUPPLEMENTARY FIGURE S1 Upper gastrointestinal series image. **(A)** No contrast extravasation. **(B)** Failure of contrast passage through gastric body and antrum (Yellow-Dashed-Line Marked Area). Aggregated dilated small bowel loops occupy the hepato-gastric space, displacing and compressing the gastric lumen (White-Dashed-Line Marked Area).

SUPPLEMENTARY FIGURE S2 Intraoperative photograph **(A)** Herniated small bowel loop compressing the pylorus. **(B)** Ischemic changes due to compression observed in the herniated small bowel and mesentery approximately 75 cm from the hernia ring. **(C)** Hernia ring corresponds to a congenital defect in the lesser omentun. **(D)** No inflammatory exudative changes detected in the lower esophagus and cardia upon exploration. **(E)** Gastric perforation ruled out by “inflation test”. **(F)** Beyond the hernia ring, a thinned area is visible in the lesser omentum (Dashed-line Marked Area).

SUPPLEMENTARY FIGURE S3 Mechanism flowchart of pneumomediastinum secondary to lesser sac hernia.
